# SEOM clinical guidelines in gestational trophoblastic disease (2017)

**DOI:** 10.1007/s12094-017-1793-0

**Published:** 2017-11-17

**Authors:** A. Santaballa, Y. García, A. Herrero, N. Laínez, J. Fuentes, A. De Juan, V. Rodriguez Freixinós, J. Aparicio, A. Casado, E. García-Martinez

**Affiliations:** 1 Servicio de Oncología Médica, Hospital Universitari i Politècnic La Fe, Valencia, Spain; 2 Servicio de Oncología Médica, Corporació Sanitària Parc Taulí, Sabadell, Spain; 3 Servicio de Oncología Médica, Hospital Miguel Servet, Saragossa, Spain; 4 Servicio de Oncología Médica, Complejo Hospitalario de Navarra, Pamplona, Spain; 5 Servicio de Oncología Médica, Hospital Nuestra Señora de Valme, Seville, Spain; 6 Servicio de Oncología Médica, Hospital Universitario Marqués de Valdecilla, Santander, Spain; 7 Servicio de Oncología Médica, Hospital Universitario Vall d´Hebrón, Barcelona, Spain; 8 Servicio de Oncología Médica, Hospital Clínico Universitario San Carlos, Madrid, Spain; 9 Servicio de Oncología Médica, Hospital Universitario Morales Meseguer, Murcia, Spain

**Keywords:** Gestational trophoblastic disease, Gestational trophoblastic neoplasia, Chemotherapy

## Abstract

Gestational trophoblastic disease (GTD) is a rare but curable disease. Recent improvements in diagnosis and molecular biology have resulted in changes in staging and treatment. These guidelines provide evidence-based recommendation on how to manage GTD.

## Introduction

Gestational trophoblastic disease (GTD) comprises a group of disorders that arise from placental trophoblastic tissue after abnormal fertilization and may follow a hydatidiform mole or a nonmolar pregnancy. These disorders include premalignant and malignant conditions. Complete hydatidiform mole (CHM) and partial hydatidiform mole (PHM) represent premalignant condition. The malignant forms of the disease are known as gestational trophoblastic neoplasia (GTN). GTN is comprised of four histologic subtypes: invasive mole (IM), choriocarcinoma (CC), epithelioid trophoblastic tumor (ETT), and placental-site trophoblastic tumor (PSTT).

Its incidence in Spain is difficult to establish, because there are no data collected from pregnancies that degenerate into GTN. The majority (80%) of GTDs are hydatidiform moles, 15% are invasive moles, and 5% are CC [1]. Approximately 50% of GTN cases result from a molar pregnancy, 25% from spontaneous abortions, and another 25% from viable pregnancies. Postmolar GTN develops in approximately 15–20% of patients after a CHM and in 5% after a PHM. The incidence after a nonmolar pregnancy ranges from 2 to 7 per 100,000 pregnancies [2].

The best established risk factor is advanced maternal age (> 40 years). The previous molar pregnancy is the second best established risk factor for hydatidiform mole and CC. History of spontaneous or induced abortions and maternal A or AB blood group have a weaker relationship with GTD.

## Methodology

SEOM guidelines have been developed with the consensus of ten medical oncologists from GEICO and SEOM. To assign level of certainty and grade of recommendation, the United States Preventive Task Force guidelines methodology was selected as reference. The final text has been reviewed and approved by all authors.

## Pathology

All forms of GTD are derived from components of the placenta, and represent abnormal counterparts of the villous and extravillous (interstitial) trophoblast.

### Complete and Partial Hydatidiform Moles

Hydatidiform moles (HMs) arise from villous trophoblast. Characteristically, first-trimester CHMs show abnormal villous, grape-like structure with diffuse trophoblast hyperplasia, stromal hypercellularity, and collapsed villous blood vessels. By contrast, the early PMHs demonstrate a patchy villous hydrops hyperplasia with scattered abnormally irregular villi and trophoblastic pseudoinclusions [3]. Diagnostic methods include the nuclear immunostaining for p57KIP2 of cytotrophoblast and villous mesenchyme in placenta which is only expressed by the maternal genome, and, therefore, is positive in PHM or nonmolar pregnancies. In addition, ploidy analysis by in situ hybridization and/or flow cytometry can distinguish PHM (triploid conceptions) from CHM and nonmolar hydropic abortions. Invasive mole has been defined as a malignant form of GTD in which, CHM or, less frequently a PHM, invades the myometrium and is associated with persistent human chorionic gonadotropin (hCG) elevation after molar evacuation. Unfortunately, there are no histopathologic features that reliably predict which patients will develop persistent GTD (pGTD)/GTN, and hence, all HMs require hCG surveillance. Of note, invasive mole can be distinguished from gestational CC by the presence of chorionic villi.

### Choriocarcinoma

CCs are malignant hCG-producing tumors arising from villous trophoblast that express human placental lactogen (hPL). CCs are characterized by invasion of the myometrium, specific trophoblastic hyperplasia and anaplasia, absence of formed chorionic villi, and hemorrhage with central necrosis. Approximately 25% of cases follow abortion or tubal pregnancy, 25% are associated with term or preterm gestation, and the remaining 50% arise from HMs. Although only 2–3% of HMs are estimated to progress to CCs. Intraplacental CC is rarely discovered, probably because placentas are not routinely sent for pathology review, so their true incidence is unknown. When it is discovered is probably the source of metastatic disease after term pregnancies.

### Placental-site trophoblastic tumor/epithelioid trophoblastic tumor

Placental-site trophoblastic tumors arise from the placental implantation site which are characterized by monomorphic infiltrating nests and sheets of interstitial trophoblasts associated with less vascular invasion, hemorrhage, necrosis, and lower hCG concentrations than does CC. PSTT commonly involve lymphatic nodes and immunohistochemistry is positive for hPL. ETT is a rare variant of PSTT, with similar clinical behavior but distinctive hyaline-like matrix.

## Molecular biology

Almost 80% of CHM are diploid (46,XX) resulting from duplication of the haploid genome of a single sperm after fertilization of an ovum in which the maternal chromosomes are lost during meiosis or as a consequence of postzygotic diploidization of a triploid conception. Approximately 20% of CHMs arise by dispermic fertilization of an ovum and may be 46,XX or 46,XY [4]. Of note, although, in CHMs, all chromosomes are paternally derived, mitochondrial DNA remains maternal in origin [4]. Partial hydatidiform moles are generally triploid (69,XXX, 69,XXY, or 69,XYY) as a result of fertilization of an apparently normal ovum by two sperms or occasionally a diploid sperm [4] and most reported cases of diploid PHM represent misdiagnosed CHM, hydropic abortions, or twin pregnancies. Cytogenic studies have revealed that PSTTs are more often diploid than aneuploidy, and commonly follow nonmolar gestations [5]. Genotype of GTN is also particularly helpful in patients with multiple pregnancies, since the interval from the causative pregnancy to the time of GTN diagnosis carries prognostic information.

## Diagnosis

While a plethora of symptoms and signs has historically been associated with molar pregnancy (hyperemesis, anaemia, pre-eclampsia, excessive uterine size, hyperthyroidism, and respiratory distress), such events are becoming less common due to routine ultrasonography (US) in early pregnancy leading to early diagnosis of molar pregnancy. CHM and PHM most commonly present with unexpected vaginal bleeding in the first trimester and subsequent abnormal findings on US. GTN has a varied clinical presentation depending upon the antecedent pregnancy, extent of disease, and histologic type.

### hCG measurement

An elevated hCG is often the first evidence of possible GTN.

Human chorionic gonadotropin is a heterogeneous molecule, produced by trophoblastic tissue. HCG comprises an alpha subunit common to all glycoprotein hormones including luteinising hormone (LH) and thyroid-stimulating hormone (TSH) and a specific beta subunit. Assays to detect hCG use antibodies directed against the beta subunit. The sensitivity of laboratory testing has increased in the last years. In pregnancy, this subunit is usually intact and becomes hyperglycosylated particularly during the first trimester. However, in GTD, hCG can exist as a free beta subunit, nicked free beta subunit, c-terminal peptide, beta-core, or hyperglycosylated form. Commercial assays which detect all this forms should be used for hCG monitoring in GTD; otherwise, it could lead to false-negative results or false-positive results from cross reaction with heterophile antibodies. Phantom hCG syndrome refers to a persistent or mild elevation of hCG when no true hCG or trophoblastic tissue is present or false-positive results from cross reaction with heterophile antibodies and must be recognised to avoid unnecessary treatment after primary evacuation of a molar pregnancy or after successful chemotherapy for GTN. Since large heterophile antibodies are filtered out at the level of the glomerulus, the urine hCG test is negative in the setting of heterophile antibodies [1]. Therefore, urine test may be used to exclude a false-positive result (serum positive and urine negative).

Invasive mole and CC are characterized by high levels of hCG, while PSTT and ETT have low hCG levels.

### Ultrasound

A Doppler pelvic ultrasound (US) should be performed in all women with suspected GTD to confirm the absence of pregnancy, to measure the uterine size/volume, spread of disease within the pelvis, and its vascularity. Correlating clinical history with β-HCG levels and with Doppler flow study findings is essential to make the correct diagnosis [6]. Indeed, false-positive and false-negative rates are high with ultrasound, and histological examination is essential to achieve a correct diagnosis. All products of conception from nonviable pregnancies must undergo histological examination regardless of ultrasound findings.

### Staging investigations

When the diagnosis of GTN is made, patients must be evaluated for extent of disease.

Blood test should be performed to assess renal and hepatic function, peripheral blood counts, and baseline serum hCG levels. A speculum evaluation should be performed to identify vaginal metastases. All patients with GTD should have a baseline chest radiograph. A chest CT must be ordered if chest RX suggests lung metastases, but only lesions visible in RX should be scored. MRI of the brain should be obtained if a patient is found to have metastatic disease in the lung [7]. If a patient has a CC or suspected GTN following a nonmolar pregnancy, imaging should include a CT of chest and abdomen [8], MRI of brain and pelvis, and a pelvic US. PET/CT may be useful in patients with recurrent disease in whom surgical resection is being considered [9].

## Staging and risk categorization

Staging for GTD defines prognostic groups identifying patients that will be probably cured with single-agent chemotherapy or if more aggressive treatment should be the initial choice. A patient with a score 0–6 will probably respond to single-agent chemotherapy, but a > 6 score indicates the need of combination chemotherapy. Special consideration must be taken to patients scoring 4–6 and further evaluation of other possible additional risk factors may guide the initial treatment choice. Pulsatility index evaluated with Doppler ultrasonography measures uterine vascularity and may predict MTX-resistant disease [10] and, although not widely adopted, nomograms to evaluate the decline of hCG and associated chemotherapy resistance can be used [11]. The staging system of the International Federation of Gynecology and Obstetrics (FIGO), developed from the World Health Organization (WHO) scoring system, is the most widely used system (Table 1) [12].

**Table 1:** FIGO staging system for gestational trophoblastic disease and modified WHO prognostic scoring system as adapted by FIGO

FIGO staging system for gestational trophoblastic disease	
Stage I	Disease confined to the uterus
Stage II	GTD extends outside of the uterus, but is limited to the genital structures
Stage III	GTD extends to the lungs, with or without genital tract involvement
Stage IV	All other metastatic sites

Modified WHO prognostic scoring system as adapted by FIGO				
Risk factor	Score			
	0	1	2	4
Age (years)	< 40	≥ 40	–	–
Antecedent pregnancy	Mole	Abortion	Term	–
Interval (months)*	4	4–6	7–12	> 12
Pretreatment serum hCG (mIU/mL)	<10^3^	10^3^–10^4^	10^4^–10^5^	> 10^5^
Largest tumor (including uterus)	<3 cm	3–4 cm	≥ 5 cm	–
Site of metastases	Lung	Spleen, kidney	GI tract	Brain, liver
Number of metastases	–	1–4	5–8	> 8
Prior failed chemotherapy	–	–	Single drug	≥ 2 drug

The stage should be followed by the sum of the risk factors (e.g., III:5)

For patients diagnosed of PSTT/ETT, the WHO scoring system is not valid, due to less hCG production and different clinical behavior and response to chemotherapy, but they should be staged with the FIGO staging system.

## Treatment

Suction and curettage under ultrasound control are mandatory in the first step of treatment of the HM. Medical induction of labor or hysterectomy is not recommended due to an increased risk for developing postmolar GTN requiring chemotherapy [13].

Hydatidiform moles of gestational age greater than 16 weeks should be evacuated at a center with experience in the GTNs management because of the risk for pulmonary embolization of molar tissue.

Criteria to initiate chemotherapy following the diagnosis of GTD include [7]:

- plateaued or rising hCG after evacuation or,
- histological evidence of choriocarcinoma or,
- metastatic disease to the brain, liver or gastrointestinal tract or,
- lung metastasis > 2 cm or,
- serum hCG ≥ 20.000 IU/l > 4 weeks after evacuation (risk of uterine perforation) or,
- heavy vaginal bleeding or any intraperitoneal or gastrointestinal hemorrhage.

### Low-risk disease

The vast majority (about 95%) of GTN is low risk (FIGO stage I or score 0–6).

In stage I, the role of second suction and curettage under ultrasound control in decreasing the chemotherapy´s need is controversial [7]. A primary hysterectomy may be considered for perimenopausal patients without the desire to preserve fertility, ideally resulting in a reduction of administered cycles of chemotherapy and a subsequent reduction of toxic effects [14, 15]. In stage IV, residual lesions after chemotherapy are not predictive of an increased risk for recurrence, so surgical resection is not indicated [16].

The most common chemotherapy regimens used are methotrexate (MTX), with or without folinic acid (FA), and actinomycin-D (ActD), but there are no consensus regarding the best single treatment (Table 2) [17]. Some retrospective and nonrandomized studies with different doses, schedules, and criteria to select patients have shown a 50–90% complete remission. A phase III trial demonstrated that the biweekly ActD regimen has a higher complete response rate than the weekly methotrexate regimen in low-risk GTN [18]. However, patients failing the first-line therapy can be easily salvaged with the second-line (or occasionally third-line) and in the end, all low-risk GTN patients achieved remission regardless of their initial response with a nearly 100% survival. For that reason and its favourable toxicity profile, the preferred first-line regimen is usually MTX with FA rescue (“eight-day regimen) [19, 20]. There are some phase III trial comparing MTX and ActD chemotherapy ongoing. Chemotherapy for low-risk disease should be continued for 6 weeks after hCG normalization.

**Table 2:** Chemotherapy schemes

Low risk	High risk
Preferred regimen	Preferred regimen
Methotrexate (MTX)^a^ 50 mg by intramuscular infection repeated every 48 h for a total of four doses Folinic acid^a^ 15 mg orally 30 h after each injection of MTX	EMA-CO Day 1: actinomycin-D 0.5 mg iv, etoposide 100 mg/m^2^ iv, methotrexate 300 mg/m^2^ iv Day 2: actinomycin-D 0.5 mg iv, etoposide 100 mg/m^2^ iv, folinic acid 15 mg post 12 hourly × 4 doses Day 8: vincristine 0.8 mg/m^2^ (maximum 2 mg), cyclophosphamide 600 mg/m^2^
Alternative regimens with methotrexate	Other regimens
Methotrexate 30–50 mg/m^2^ intramuscular (IM) weekly	EP/EMA Day 1: actinomycin-D 0.5 mg iv, etoposide 100 mg/m^2^ iv, methotrexate 300 mg/m^2^ iv Day 2: folinic acid 15 mg 12 hourly × 4 doses. Starting 24 h after methotrexate week 2 Day 8: etoposide 150 mg/m^2^ (maximum 2 mg), cisplatin 75 mg/m^2^
Methotrexate 0.3–0.5 mg/kg intravenous (IV) or IM daily for 5 days every 2 weeks (maximum 25 mg per dose)	
Methotrexate MTX 100 mg/m^2^ IV over 30 min followed by MTX 200 mg/m^2^ IV infusion over 12 h Folinic acid 15 mg every 12 h in six doses im or orally beginning 24 h after starting MTX	
Alternative regimens with actinomycin-D	
Actinomycin-D 10–12 µg/kg IV push daily for 5 days	
Actinomycin-D 1.25 mg/m^2^ iv push every 2 weeks	

^a^Courses repeated every 2 weeks

Primary resistance is defined as an increase or a plateau in two consecutive hCG values during single-agent chemotherapy and occurs in 10–30% of patients with low-risk GTN. If levels of hCG are low phantom, hCG syndrome must be excluded. The second-line single-agent chemotherapy usually is the one that has not been used before: patients treated with MTX, received biweekly bolus ActD. If the initial treatment was ActD, then the treatment with MTX with or without FA is prescribed [20]. However, patients with higher risk scores (5–6) are at a greater risk of resistant disease (30–50%) compared with those with lower prognostic scores. For such patients, multi-agent chemotherapy is a reasonable option. Patients who have resistant or recurrent disease despite second-line single-agent chemotherapy (15% of the patients) should be treated with combination chemotherapy (see treatment of high-risk disease). For patients who do not respond to the initial combination chemotherapy, alternative regimens or surgery can be offered.

### High-risk disease

Patients with an FIGO score of ≥ 6 have a high risk of developing drug resistance and should be treated multi-agent chemotherapy. Several combinations have been developed (Table 2) including: MTX, FA, and ActD (MFA); MTX, ActD, cyclophosphamide, doxorubicin, melphalan, hydroxyurea, and vincristine (CHAMOCA); MTX, ActD and cyclophosphamide (MAC); etoposide, MTX, and ActD (EMA) and FAV (5-FU, actinomycin-D, and vincristine [7]. EMA/CO (etoposide, methotrexate, actinomycin-D plus cyclophosphamide, and vincristine) is currently the most widely used first-line combination chemotherapy, because it is effective with predictable and easily managed short-term toxicity, although this regimen has not been rigorously compared to other combinations such as MAC or FAV in randomized trials. CHAMOCA is not recommended for GTN treatment as it is more toxic and not more effective than MAC [21].

Therapy should be continued for 6 weeks of normal hCG values or 8 weeks if poor prognostic features such as liver or brain metastases were present.

A high FIGO score (> 13) is associated with poor survival, where death is not only linked to chemoresistance but also to early and severe complications, such as hemorrhagic metastases, infection, multisystem organ failure, or tumor lysis syndrome. Appropriate and rapid diagnosis, treatment by specialized centers, and reduction of early deaths because of chemotherapy initiation have led to significant improvements in survival for patients with high-risk. Low-dose EP (etoposide 100 mg/m^2^ and cisplatin 20 mg/m^2^ on days 1 and 2 repeated weekly) for 1–2 cycles before commencing EMA-CO, was shown to decrease early death rate from 11 of 140 (7.2%) to 1 of 140 (0.7%) [22].

### Resistant or recurrent disease

Up to 20–25% of women with high-risk metastatic GTN have disease persistence, progress on, or relapse after primary chemotherapy. However, with the current salvage therapies, cure is still possible in more than 75% of cases. Both surgical resection and alternate cisplatin-based regimens should be considered [1]. Secondary hysterectomy and metastasectomy (i.e., pulmonary resection, craniotomy, and liver lobe resection) play a significant role in the management of chemo-resistant disease [23].

These patients benefit from the identification of isolated active disease sites amenable for surgical resection. In one series, surgical resection of chemo-resistant disease was possible in 39% of cases, with long-term survival of 82% [24]. If complete surgical removal is not feasible or BHCG levels fail to decline adequately after resection, alternate chemotherapy regimens are needed. Cisplatin, paclitaxel, gemcitabine, and capecitabine are active drugs in this setting. The most commonly used schedule is EMA-EP (etoposide, methotrexate, and dactinomycin alternating weekly with etoposide plus cisplatin) [25], resulting in a response rate of 75–80%. Some evidence suggests that TE/TP (paclitaxel and etoposide alternating 2 weekly with paclitaxel and cisplatin) [26] is as effective and less toxic than EMA-EP. A randomized trial comparing both combinations is ongoing. Other regimens investigated in this setting are ACE (dactinomycin, cisplatin, and etoposide), VIP (etoposide, ifosfamide, and cisplatin), BEP (bleomycin, etoposide, and cisplatin), and ICE (high-dose ifosfamide, carboplatin, and etoposide), but outcome data with them are scant. Experience with high-dose chemotherapy (carboplatin and etoposide-based) with peripheral stem-cell support is very limited.

Palliative surgery is needed in case of life-threatening hemorrhage no responding to selective angiographic embolization [19].

### Placental-site trophoblastic tumor and ETT

The primary treatment in patients with PSTT is surgical. Hysterectomy with pelvic lymph node sampling is recommended for stage I disease presenting within 4 years of the last known pregnancy. Residual masses are also removed surgically.

Patients with metastatic disease require combination chemotherapy. EP/EMA is recommended continued for 8 weeks of normal hCG levels.

ETT is more aggressive, but the treatment is the same as PSTT.

## Follow-up

After primary surgical treatment in HM, weekly serum hCG assays should be obtained until 3 consecutive weekly assays are normal. Thereafter, in PM, the patient can be discharged from follow‐up. If repeated suction curettage will be necessary, patients should be monitored until 3 consecutive weekly serum hCG are normal and so with monthly serum hCG levels for 6 months. In CM, patients should be monitored with monthly serum hCG levels for 6 months.

Follow-up recommendations during treatment and after hCG normalization in GTN are listed in Table 3.

**Table 3:** Follow-up recommendations for gestational trophoblastic disease

Serum hCG	Hydatidiform moles	Gestational trophoblastic neoplasia	
During treatment	After primary surgical treatment: weekly serum hCG assays until 3 consecutive weekly assays are normal	Weekly serum hCG assays until 3 consecutive weekly assays are normal	
After treatment	Monthly serum hCG levels for 6 months	GTN low risk Monthly for 12 months. Every 6 months 1 year Annually until 5 years	GTN high risk Monthly for 18 months. Every 6 months 2 years. Annually until 5 years

There is still a lack of consensus in the literature about the follow-up of patients with PSST/ETT. PSTT/ETT has less hCG production, slower growth, and late metastasis, and therefore, the recommendation is to follow up for a minimum of 5 years.

**Fig. 1:**
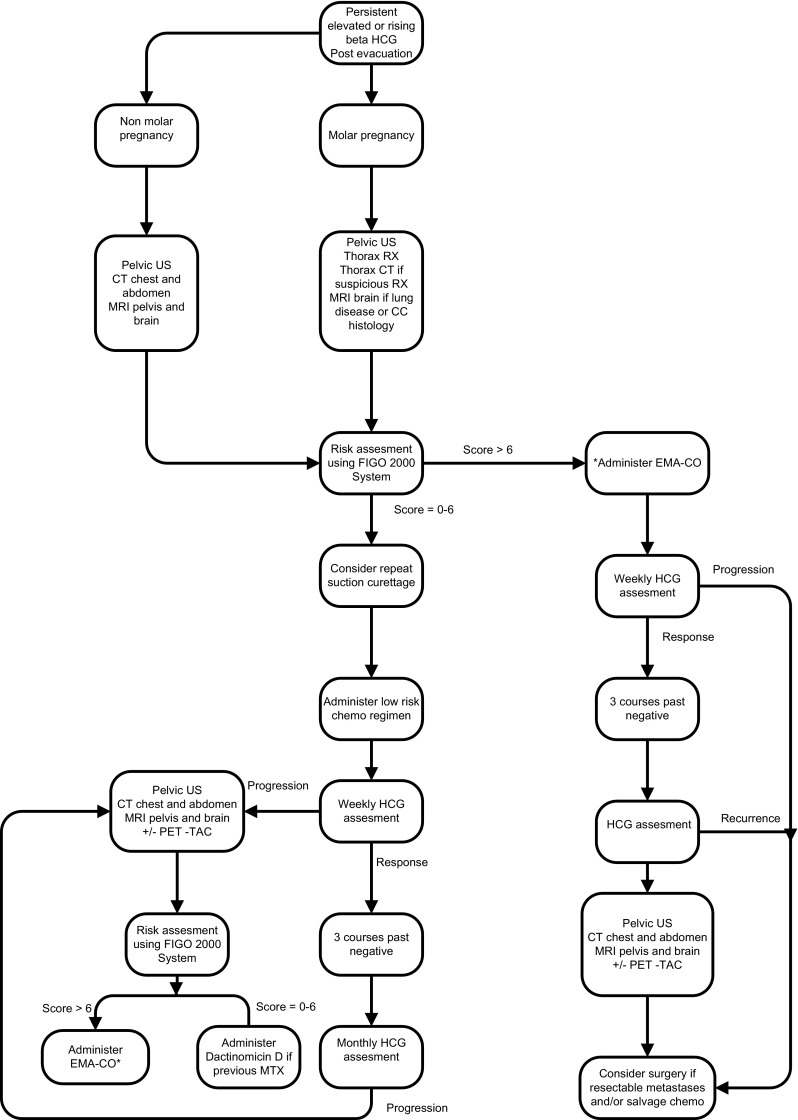
Algorithm for the management of GTD modified from Brown [1]

Management of GTD are summarized in Fig. 1.

## Contraception and fertility preservation

Contraception during follow-up relates to the importance of hCG surveillance and not the risk of recurrence. Contraception with oral contraceptives should be recommended for 6 month in hydatiform mole. After QT, in GTN, it is recommended to avoid pregnancy for 12 months in low-risk patients and for 18 months in high-risk patients. Intrauterine devices are not recommended because of the potential for uterine perforation [1].

**Table 4:** SEOM guideline recommendations for GTD

Diagnosis A serum quantitative hCG should be assessed in all women with a diagnosis of molar pregnancy and in any woman of childbearing age who presents with abnormal bleeding or has unexplained metastatic disease [IV, A] Women with GTN should have hCG, pelvic US, and chest X-ray [IV, A] If the pregnancy was no molar or if metastases are present in chest X-ray, a CT scan and an MRI of the brain should be performed [IV, A]
Staging The FIGO scoring system should be use the staging after molar pregnancies and CC, but it is not valid in PSTT/ETT [IV, A]
Treatment Suction and curettage under ultrasound control are mandatory in the first step of treatment of the HM. Medical induction of labor or hysterectomy is not recommended due to an increased risk for developing postmolar GTN requiring chemotherapy [IV, A] Criteria to initiate chemotherapy following the diagnosis of GTD include: – plateaued or rising hCG after evacuation or, – histological evidence of choriocarcinoma or, – metastatic disease to the brain, liver or gastrointestinal tract or, – lung metastasis > 2 cm or, – serum hCG ≥ 20,000 IU/l > 4 weeks after evacuation (risk of uterine perforation) or, – heavy vaginal bleeding or any intraperitoneal or gastrointestinal hemorrhage Low-risk patients should be treated with a single-agent CT. The preferred first-line regimen is MTX with FA rescue. CT should be continued for 6 weeks after hCG normalization [II, A]. A primary hysterectomy may be considered for perimenopausal patients without the desire to preserve fertility High-risk patients should be treated with multi-agent CT [IV, A]. EMA-CO is the preferred option. In patients with FIGO score > 13, low-dose EP CT can be used to reduce the early deaths [IV, A] Salvage therapy for resistant or recurrent GTN is effective in a majority of cases. Surgical resection can cure a proportion of patients with isolated sites of disease. All other cases should be treated with cisplatin-based chemotherapy schedules as EMA/EP or TE/TP [IV, A]
Follow-up During treatment in GTN, serum hCG levels should be monitored weekly to determine response until 3 consecutive normal levels [IV, A] Afterwards a monthly determination of serum hCG (for 12 months in low-risk patients and for 18 months in high-risk patients), c/6 month during 1 year in low-risk and 18 months in high-risk patients, annually until 5 years follow-up is recommended [IV, A] No follow-up imaging tests are required [IV, A]

83% of patients treated with MTX/FA or EMA/CO chemotherapy become pregnant [1]. All recommendations for the diagnosis, treatment and management of GTD are summarized in Table 4.
